# Effects of the WHO Labour Care Guide on cesarean section in India: a pragmatic, stepped-wedge, cluster-randomized pilot trial

**DOI:** 10.1038/s41591-023-02751-4

**Published:** 2024-01-30

**Authors:** Joshua P. Vogel, Yeshita Pujar, Sunil S. Vernekar, Elizabeth Armari, Veronica Pingray, Fernando Althabe, Luz Gibbons, Mabel Berrueta, Manjunath Somannavar, Alvaro Ciganda, Rocio Rodriguez, Savitri Bendigeri, Jayashree Ashok Kumar, Shruti Bhavi Patil, Aravind Karinagannanavar, Raveendra R. Anteen, Pavithra Mallappa Ramachandrappa, Shukla Shetty, Latha Bommanal, Megha Haralahalli Mallesh, Suman S. Gaddi, Shaila Chikkagowdra, Bellara Raghavendra, Caroline S. E. Homer, Tina Lavender, Pralhad Kushtagi, G. Justus Hofmeyr, Richard Derman, Shivaprasad Goudar

**Affiliations:** 1 Maternal, Child and Adolescent Health Program, Burnet Institute, Melbourne, Victoria Australia; 2 Women’s and Children’s Health Research Unit, Jawaharlal Nehru Medical College, KLE Academy of Higher Education and Research, Belgaum, India; 3 Instituto de Efectividad Clínica y Sanitaria (IECS-CONICET), Buenos Aires, Argentina; 4 Gadag Institute of Medical Sciences, Gadag, India; 5 General Hospital, Gokak, Belgaum, India; 6 JJM Medical College, Davanagere, India; 7 Vijayanagar Institute of Medical Sciences (VIMS), Ballari, India; 8 Department of International Health, Liverpool School of Tropical Medicine, Liverpool, UK; 9 Manipal Academy of Higher Education, Manipal, India; 10 Department of Obstetrics and Gynaecology, University of Botswana, Gaborone, Botswana; 11 University of the Witwatersrand and Walter Sisulu University, East London, South Africa; 12 Thomas Jefferson University, Philadelphia, PA USA

**Keywords:** Health care, Outcomes research

## Abstract

Cesarean section rates worldwide are rising, driven by medically unnecessary cesarean use. The new World Health Organization Labour Care Guide (LCG) aims to improve the quality of care for women during labor and childbirth. Using the LCG might reduce overuse of cesarean; however, its effects have not been evaluated in randomized trials. We conducted a stepped-wedge, cluster-randomized pilot trial in four hospitals in India to evaluate the implementation of an LCG strategy intervention, compared with routine care. We performed this trial to pilot the intervention and obtain preliminary effectiveness data, informing future research. Eligible clusters were four hospitals with >4,000 births annually and cesarean rates ≥30%. Eligible women were those giving birth at ≥20 weeks’ gestation. One hospital transitioned to intervention every 2 months, according to a random sequence. The primary outcome was the cesarean rate among women in Robson Group 1 (that is, those who were nulliparous and gave birth to a singleton, term pregnancy in cephalic presentation and in spontaneous labor). A total of 26,331 participants gave birth. A 5.5% crude absolute reduction in the primary outcome was observed (45.2% versus 39.7%; relative risk 0.85, 95% confidence interval 0.54–1.33). Maternal process-of-care outcomes were not significantly different, though labor augmentation with oxytocin was 18.0% lower with the LCG strategy. No differences were observed for other health outcomes or women’s birth experiences. These findings can guide future definitive effectiveness trials, particularly in settings where urgent reversal of rising cesarean section rates is needed. Clinical Trials Registry India number: CTRI/2021/01/030695.

## Abstract (web summary)

To evaluate the effects of implementing the WHO Labour Care Guide on cesarean section rates, as compared to routine intrapartum care, a pilot trial conducted in India generated preliminary evidence around the effectiveness of the strategy that can inform larger definitive trials.

## Main

An estimated 287,000 maternal deaths, 2.4 million neonatal deaths and 1.9 million stillbirths occur each year, the vast majority of which take place in low- and middle-income countries^1–3^. As many as 45% of these maternal deaths, stillbirths and neonatal deaths occur during labor, birth and the first 24 hours postpartum^4^. Ensuring good-quality care is available to all women during labor and birth (that is, the intrapartum period) is thus critical to any efforts to reduce global maternal and newborn morbidity and mortality^5^.

By 2030, an estimated 38 million women annually (28.5% of births worldwide) will undergo a cesarean section^6^. A cesarean section is an essential component of good-quality intrapartum care, yet it carries inherent risks for women and newborns^7,8^. When it is performed for a medical indication, these risks are outweighed by the benefits of intervening; it is lifesaving in some clinical situations^6,9^. However, the global cesarean rate increased by 19 percentage points between 1990 to 2018, driven in large part by cesareans performed without a clear medical indication (that is, an unnecessary cesarean section)^6^. These women and babies are exposed to the risks of cesarean sections, for no health benefit.

The World Health Organization (WHO) has long recommended that a woman in labor should be monitored by a skilled healthcare provider using a partograph, a paper clinical tool for documenting observations and helping make clinical decisions^10^. When completed prospectively, the partograph can determine whether and when an intervention—such as labor augmentation or cesarean section—is warranted. A WHO-led 1994 trial showed that prospective partograph use combined with intensive provider training optimized the use of intrapartum interventions and improved maternal and newborn outcomes^11^. Consequently, the WHO simplified partograph was widely adopted as a key component of routine intrapartum care internationally^12^. However, while more women than ever are giving birth in health facilities^13^, partographs are often used poorly, or not at all. Inadequate provider training and skills, heavy staff workloads, a lack of clinical equipment and supplies, and restrictive hospital policies are known barriers to partograph use^14–16^.

In 2018, the WHO published 56 recommendations to improve the quality of intrapartum care and enhance women’s childbirth experiences^17^. Key recommendations included changing the definition of active first stage of labor from the widely used 3 cm or 4 cm to starting from 5 cm of cervical dilation, and removal of the ‘alert’ and ‘action’ lines. These changes reflected a growing body of evidence that the historical ‘1 cm per hour’ rule for active labor progress is unrealistic for most women, and that slower dilation rates are not associated with adverse birth outcomes. In response to these recommendations, a ‘next generation’ partograph known as the WHO Labour Care Guide (LCG) was developed in 2020 through expert consultations, primary research with maternity healthcare providers and a multicountry usability study^18–20^.

The LCG aims to promote the use of evidence-based, respectful and woman-centered care during labor and childbirth^21^. The healthcare provider regularly records clinical parameters related to labor progress and maternal and fetal wellbeing; deviations from normal are highlighted to ensure the required actions are taken. The LCG has specific, evidence-based time limits for each centimeter of cervical dilatation. The provider also documents the provision of important, yet often overlooked, supportive care practices—labor companionship, oral hydration, mobility during first stage, birth position of choice and pain management. It is also used for monitoring the second stage of labor.

The WHO states that the LCG should be implemented into routine care globally^22^. However, introducing the LCG requires an active strategy that ensures a healthcare provider’s clinical practice improves, thereby enhancing the quality of intrapartum care, reducing the use of unnecessary interventions and improving support to women during labor. However, as the LCG is a novel tool, no such strategy has been developed or tested in a randomized trial. This knowledge gap was highlighted in the WHO’s recent global LCG research prioritization exercise, in which identifying optimal implementation strategies, as well as understanding the LCG’s effects on maternal and perinatal outcomes, were top research priorities^23^.

To address this gap, we conducted formative research and developed a complex ‘LCG strategy’ intervention. The intervention included a co-designed LCG training program for providers working in labor wards, comprising initial and refresher training workshops and 8 weeks of case-based teaching sessions using the LCG. The LCG strategy also included implementing monthly audit and feedback meetings for the hospital’s birth and cesarean data. These data were reported using the ten group Robson classification system, which is recommended by the WHO for evaluating cesarean section use^9^. It classifies women into one of ten groups on the basis of their parity, whether previous cesarean was used or not, onset of labor, fetal presentation and lie, number of neonates and gestational age (term or preterm)^24^. For example, women in Robson Group 1 are those who are nulliparous, gave birth to a singleton, term pregnancy in cephalic presentation, and were in spontaneous labor. Robson Group 1 usually accounts for 30% of the obstetric population, and overuse of cesarean section is often observed in this group.

In this pilot trial, we aimed to evaluate the effects of implementing the LCG strategy, as compared to routine intrapartum care; the latter included use of the simplified partograph. We performed this pilot trial to demonstrate whether the LCG strategy was practicable, as well as to generate preliminary effectiveness evidence to inform future research.

## Results

### Characteristics of study population

**Fig. 1:**
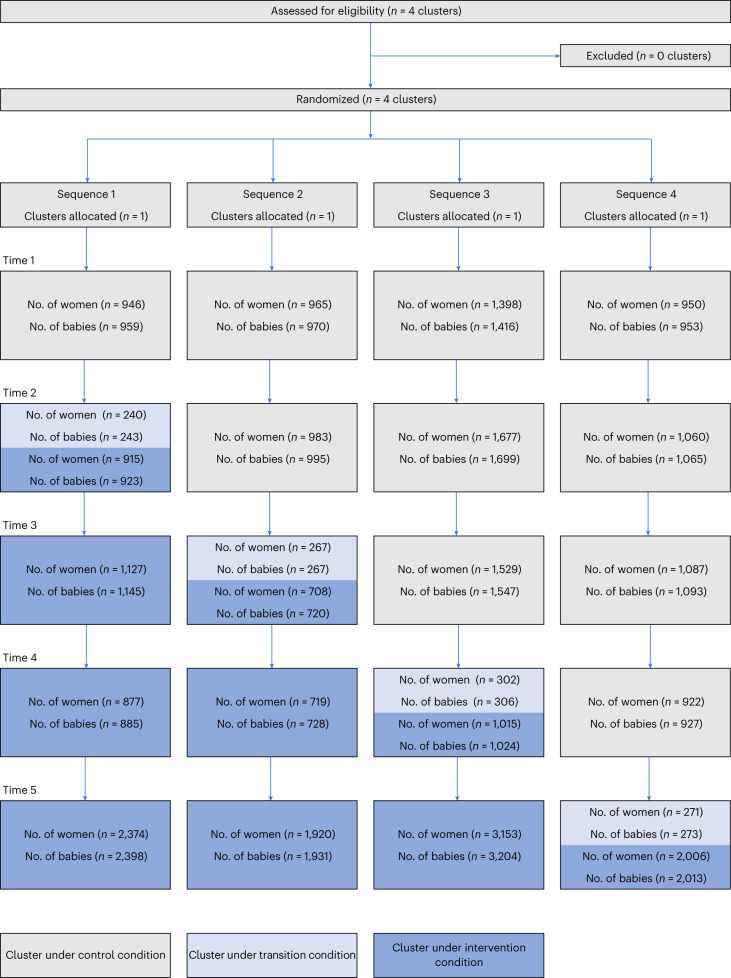
Trial diagram showing the number of women with a gestational age ≥20 weeks by hospital and time period. The four clusters (hospitals) were randomly allocated to four different sequences. Each sequence had a different schedule of control condition (gray), transition condition (light blue) and intervention condition (dark blue). Each cell shows the number of women and babies at each time point.

Between 1 July 2021 and 15 July 2022, 26,331 women gave birth to 26,595 babies in the four hospitals during the control and intervention periods and were included for analysis (Fig. 1). The total number of women giving birth differed between hospitals, ranging from 5,295 to 8,772 women per hospital. The analysis population comprised 11,517 women (11,624 babies) who gave birth in the control period and 14,814 women (14,971 babies) who gave birth in the intervention period. The main analysis did not include the 1,080 women (1,089 babies) who gave birth in the transition period. Clusters implemented the intervention at the scheduled time, with no substantive adaptations.

While there were more women in the intervention than the control, the characteristics of women were similar (Table 1). Nearly half of included women were nulliparous (46.7% of the control group and 47.5% of the intervention group), while more than half of multiparous women had no prior cesarean section (56.7% versus 55.0%). The distribution of women across the ten Robson classification groups was also similar (Supplementary Table 1). Robson Group 1 accounted for 30.8% (3,543 of 11,517) of women in the control group and 29.0% (4,302 of 14,814) of women in the intervention group. The intervention group had a slightly higher proportion of women in Group 2 and a slightly lower proportion of women in Group 3.

**Table 1:** Characteristics of the study population

Characteristic	Intervention period (*N* = 14,814 women)	Control period (*N* = 11,517 women)
	*n* (%)	*n* (%)
Maternal age (years)^a^	23.9 (3.6)	23.4 (3.6)
Maternal age		
Less than 20	1,020 (6.9)	1,010 (8.8)
20–34	13,572 (91.6)	10,357 (89.9)
35 or more	222 (1.5)	150 (1.3)
Previous cesarean section^b^		
0	4,282 (55.0)	3,484 (56.7)
1	2,819 (36.2)	2,133 (34.7)
2 or more	682 (8.8)	525 (8.5)
Gravida		
1	6,394 (43.2)	4,940 (42.9)
2–4	8,160 (55.1)	6,369 (55.3)
5 or more	260 (1.8)	208 (1.8)
Parity		
0	7,031 (47.5)	5,375 (46.7)
1–3	7,674 (51.8)	6,022 (52.3)
4 or more	109 (0.7)	120 (1.0)
Women receive antenatal care during pregnancy	14,745 (99.5)	11,438 (99.3)
Covid status at admission		
Positive	32 (0.2)	5 (0.0)
Negative	8,208 (55.4)	9,168 (79.6)
Pending or not done	6,574 (44.4)	2,344 (20.4)
Transferred from another health facility during labor	2,102 (14.2)	1,881 (16.3)
Gestational age at time of birth^a^	38.3 (2.5)	38.3 (2.6)

^a^Mean and standard deviations are reported.

^b^Multiparous women only were considered.

### Primary and secondary outcomes

Table 2 reports the intervention effect sizes for the primary outcome and secondary maternal process-of-care outcomes. For the primary outcome, the cesarean section rate in Robson Group 1 for the control group was 45.2%, while in the intervention group it was 39.7%, with a crude absolute difference of −5.5% (relative risk (RR) 0.85, 95% confidence interval (CI) 0.54–1.33, *P* value 0.1088). The estimated intraclass correlation coefficient (ICC) for the primary outcome during the control period was 0.015 (95% CI 0; 0.043). For secondary outcomes, the cesarean section rate in Robson Groups 1 and 3 was 30.9% for the control group, and 26.9% for the intervention group—a crude absolute difference of −4.0% (RR 0.81, 95% CI 0.59–1.11). The overall cesarean section rate was 50.5% for the control group and 50.7% for the intervention group (RR 0.91, 95% CI 0.71–1.15) For the secondary outcome augmentation with oxytocin during spontaneous labor, the prevalence in the control group was 27.3% and in the intervention group it was 9.3% (crude absolute difference −18.0%). However, the estimate of effect was not significant (RR 0.34, 95% CI 0.01–15.04)—the wide CI was attributable to the high variability in outcome prevalence between hospitals and time periods.

**Table 2:** Effect of the intervention on primary outcome and maternal process-of-care outcomes

	Intervention period (*N* = 14,814 women)		Control period (*N* = 11,517 women)		RR (95% CI)^e^
	*n/N*	(%)	*n/N*	(%)	
Primary outcome					
Cesarean section in Robson Group 1	1,709/4,302	(39.7)	1,602/3,543	(45.2)	0.85 (0.54–1.33)
Maternal process-of-care outcomes					
Cesarean section in women in Robson Groups 1 and 3	2,012/7,485	(26.9)	1,919/6,204	(30.9)	0.81 (0.59–1.11)
Cesarean section in women in Robson Groups 1, 2, 3, 4 and 5	6,529/12,735	(51.3)	5,028/9,808	(51.3)	0.92 (0.78–1.10)
Cesarean section (all women)	7,505/14,814	(50.7)	5,817/11,517	(50.5)	0.91 (0.71–1.15)
Augmentation with oxytocin during labor^a^	912/9,764	(9.3)	2,273/8,318	(27.3)	0.34 (0.01–15.04)
Artificial rupture of the membranes^a,d^	553/9,764	(5.7)	559/8,318	(6.7)	-
Episiotomy^b^	4,820/7,309	(65.9)	3,137/5,700	(55.0)	0.99 (0.73–1.35)
Operative vaginal birth^b^	192/7,309	(2.63)	112/5,700	(1.96)	1.12 (0.13–9.36)
Days from admission to childbirth^c^	0.34	(0.73)	0.30	(0.68)	0.05 (−0.31–0.41)
Days from childbirth to discharge^c^	3.29	(1.75)	3.52	(1.88)	0.23 (−0.84–1.30)

^a^Women in spontaneous labor were considered.

^b^Women with vaginal deliveries were considered.

^c^The mean of the days and standard deviations are reported. The effect size was calculated as the difference between the mean of days in the intervention group and the mean of days in the control group.

^d^RR was not estimated since convergence of the model was not achieved.

^e^The RR and 95% CI were estimated with the generalized estimating equation method employing the Manck and DeRouen bias correction method and a degree of freedom approximation.

Table 3 reports the intervention effects on other secondary maternal, fetal and newborn health outcomes. For maternal secondary outcomes—third- or fourth-degree tears, postpartum hemorrhage requiring uterine balloon tamponade or surgical intervention, and maternal infection requiring therapeutic antibiotics—the prevalence was less than 1% in both groups, and there were no clear differences. For the baby, there were no clear differences in stillbirth (RR 0.97, 95% CI 0.43–2.19), neonatal death before discharge/day 7 (RR 1.31, 95% CI 0.37–4.71) or perinatal death before discharge/day 7 (RR 1.06, 95% 0.41–2.73). We measured several newborn morbidity outcomes before discharge/day 7 (Apgar score <7 at 5 minutes; use of bag and mask ventilation; use of mechanical ventilation; >48 hour admission in neonatal intensive care unit (NICU); and newborn requiring NICU admission for hypoxic ischemic encephalopathy) and found no clear differences for any of these.

**Table 3:** Effect of the intervention on maternal, perinatal and neonatal health outcomes

	Intervention period (*N* = 14,814 women)		Control period (*N* = 11,517 women)		RR (95% CI)^a^
	*n/N*	(%)	*n/N*	(%)	
Maternal secondary outcomes					
Third- or fourth-degree tears	18/14,814	(0.12)	25/11,517	(0.22)	0.51 (0.01–29.16)
Postpartum hemorrhage requiring uterine balloon tamponade or surgical intervention	28/14,814	(0.19)	46/11,517	(0.40)	0.38 (0.00–84.07)
Suspected or confirmed maternal infection requiring therapeutic antibiotics	114/14,814	(0.77)	53/11,517	(0.46)	2.12 (0.06–70.96)
Fetal/neonatal secondary outcomes					
Stillbirth	449/14,971	(3.00)	367/11,624	(3.16)	0.97 (0.43–2.19)
Antepartum stillbirth	279/14,971	(1.86)	286/11,624	(2.46)	0.91 (0.34–2.47)
Intrapartum stillbirth	163/14,971	(1.09)	79/11,624	(0.68)	0.90 (0.49–1.65)
Apgar score <7 at 5 minutes	670/14,522	(4.61)	567/11,257	(5.04)	1.17 (0.86–1.59)
Bag and mask ventilation of newborn	424/14,522	(2.92)	256/11,257	(2.27)	1.21 (0.08–18.75)
Mechanical ventilation of newborn	293/14,522	(2.02)	260/11,257	(2.31)	1.29 (0.36–4.66)
Prolonged (>48 hour) admission in NICU	1,843/14,522	(12.7)	1,014/11,257	(9.0)	1.14 (0.47–2.79)
Newborns requiring NICU admission for hypoxic ischemic encephalopathy	34/14,522	(0.23)	152/11,257	(1.35)	0.40 (0.04–3.74)
Composite neonatal morbidity outcome^b^	376/14,522	(2.59)	377/11,257	(3.35)	1.11 (0.32–3.79)
Neonatal death	200/14,522	(1.38)	196/11,257	(1.74)	1.31 (0.37–4.71)
Perinatal death (stillbirth or neonatal death)	649/14,971	(4.34)	563/11,624	(4.84)	1.06 (0.41–2.73)

^a^The RR and 95% CI were estimated with the generalized estimating equation method employing the Manck and DeRouen bias correction method and a degree of freedom approximation.

^b^The composite neonatal outcome was defined as one or more of the following: mechanical ventilation of the newborn, requirement of NICU admission for hypoxic ischemic encephalopathy of the newborn or neonatal death.

A total of 1,438 women in the control group and 1,277 women in the intervention group consented (100% and 99.9% consent rate, respectively) and completed postpartum surveys. Table 4 reports the effects on women’s experiences at birth, for which there were no differences between groups. In terms of adverse events, there were five maternal deaths, 196 neonatal deaths and 367 stillbirths in the control period, and 13 maternal deaths, 200 neonatal deaths and 449 stillbirths in the intervention period (Supplementary Tables 3 and 4). None of these deaths were assessed as being related to the intervention.

**Table 4:** Effect of the intervention on women’s experience outcomes (women in Robson Group 1 or 3)

	Intervention period (*N* = 1,277 women)		Control period (*N* = 1,438 women)		RR (95% CI)^a^
	*n/N*	(%)	*n/N*	(%)	
Women reporting labor companion	982/1,277	(76.9)	1,206/1,438	(83.9)	1.19 (0.89–1.59)
Women reporting being offered pain relief	196/1,277	(15.3)	75/1,438	(5.2)	2.30 (0.00–1,281.82)
Women reporting being very satisfied or somewhat satisfied with how their pain was managed	827/1,277	(64.8)	957/1,437	(66.6)	0.94 (0.06–16.14)
Women reporting being encouraged to drink water	863/1,277	(67.6)	1,123/1,438	(78.1)	0.98 (0.34–2.86)
Women reporting being encouraged to eat food	657/1,277	(51.4)	823/1,438	(57.2)	0.99 (0.13–7.37)
Women reporting being encouraged to walk	827/1,277	(64.8)	863/1,437	(60.1)	1.10 (0.34–3.58)
Women reporting being asked which birth position they preferred	27/1,277	(2.11)	10/1,438	(0.70)	1.96 (0.00–1,384.48)
Women reporting being very or somewhat satisfied with the amount of time the health provider spent with them	1,260/1,277	(98.7)	1,424/1,437	(99.1)	0.99 (0.93–1.05)
Women reporting being very or somewhat satisfied with the way the health provider communicated with them	1,262/1,277	(98.8)	1,424/1,438	(99.0)	0.99 (0.91–1.07)
Women who strongly agreed or agreed that their privacy was respected	1,234/1,277	(96.6)	1,315/1,438	(91.4)	0.99 (0.56–1.75)
Women who reported being asked permission before examinations	596/1,277	(46.7)	992/1,438	(69.0)	0.84 (0.07–10.34)
Women who reported being asked permission before treatments	588/1,277	(46.0)	996/1,438	(69.3)	0.85 (0.07–10.37)
Women who strongly agreed or agreed that they were satisfied with their labor and birth experience	1,268/1,277	(99.3)	1,404/1,438	(97.6)	1.01 (0.95–1.07)

^a^The RR and 95% CI were estimated with the generalized estimating equation method employing the Manck and DeRouen bias correction method and a degree of freedom approximation.

## Discussion

In this stepped-wedge, cluster-randomized pilot trial in India, we implemented a strategy to introduce the LCG into routine care for women giving birth, as well as initiating monthly audit and feedback meetings on cesarean section data using Robson classification. We observed a 5.5% crude absolute reduction in cesarean rates among women in Robson Group 1 following introduction of the intervention; however, this difference was not statistically significant. Maternal process-of-care measures were not significantly different, though the crude absolute difference for labor augmentation using oxytocin was −18.0%. We did not observe any clear differences in maternal, fetal or newborn health outcomes, or women’s experiences at birth. The findings do not preclude the possibility that the LCG strategy may reduce cesarean section and augmentation of labor in larger, definitive trials.

Reversing the worldwide trend in rising cesarean section rates, driven in large part by medically unnecessary cesarean use, has proven to be a challenging problem—a 2018 WHO guideline identified few effective interventions to address this^25,26^. The LCG promotes several supportive care measures that have been shown in trials to prevent cesarean section, such as labor companionship, mobilization during labor and adequate pain relief^27–29^. Also, the use of 5-cm dilatation to define the active first stage, as well as removal of the ‘1-cm-per-hour rule’, would, assumedly, lead to fewer intrapartum interventions. As the LCG is a novel clinical tool, there are few effectiveness studies available for comparison, though more trials using the LCG are planned^30,31^. In 2022, Pandey et al. published findings of an individually randomized trial of 271 low-risk women in a single hospital in India, comparing the effects of using the LCG versus modified partograph^32^. They reported a dramatic reduction in cesarean section—1.5% in the LCG group compared with 17.8% in the control group (*P* value 0.0001)—as well as significantly lower oxytocin use and shorter duration of the active phase of labor with the LCG.

In planning this trial, the sample size calculation was based on an estimated 25% RR reduction for cesarean section rate in Robson Group 1. The intervention was implemented as planned with good uptake, and the target sample size was met. As this was a pilot trial, we cannot draw definitive conclusions on the magnitude of the LCG strategy’s effect on the primary or secondary outcomes. However, we consider these pilot trial findings to be promising, and that further definitive trials are warranted. The trial cannot test a superiority hypothesis for rarer adverse outcomes (such as mortality and severe morbidity of women and babies), although, reassuringly, there was no evidence of short-term harms associated with the LCG strategy. Data on these outcomes should be monitored in future, larger-scale research.

We did not detect any differences for outcomes on women’s experiences. However, these data showed women had high levels of satisfaction with the amount of time health workers spent with them, with the way they were communicated with and with their overall birth experience. It also showed that some supportive care practices, such as being offered a labor companion, were reasonably common, though other women-centered interventions were not well implemented. For example, being offered pain relief (5.2% and 15.3%) and being asked which birth position they preferred (0.7% and 2.1%) were poorly used. This highlights that substantive gaps persist in the provision of supportive care around the time of birth. Additional strategies are needed to address these gaps.

This trial was conducted in large, busy, public tertiary hospitals with high cesarean use, within one state of India. In three hospitals, partograph completion was the responsibility of postgraduate residents only. In India, the national Labour Room Quality Initiative (‘LaQshya’) and hospital accreditation process^33^ has a strong emphasis on respectful maternity care, which is well aligned with the WHO’s recommendations and the LCG’s foundational principles. These factors mean the trial findings may not necessarily generalize to other settings that are naïve to respectful maternity care principles and policies. For example, it may be more challenging to generate provider behavior change in settings without a national policy framework. Contextual differences around how frequently obstetric interventions are used, as well as differences in the risk profile of obstetric populations, may mean the LCG strategy has variable effects.

We describe this study as a pilot trial as it was exploratory—we tested a complex intervention for which the effect size was initially unknown. We also demonstrated viability of the LCG strategy and the stepped-wedge study design, and generated evidence for a future definitive trial (particularly sample size). Such a trial should use a stepped-wedge, cluster-randomized design and should involve more hospitals (clusters) that have high rates of cesarean section. Such a trial would also be able to assess other, rarer adverse outcomes. Strengths include the use of a theory-based, evidence-informed, co-design approach to developing the LCG strategy, which aimed to address factors known to impair partograph use^16^. We also used a robust, cluster-randomized design and recruited a large number of participants in a real-world clinical setting. The stepped-wedge design means that all hospitals were implementing the LCG strategy at trial conclusion.

This trial nonetheless has some limitations. CIs for several outcomes were quite wide. This was driven by variability in outcome rates between time periods and between clusters, as well as the small number of clusters. Also, as this is a pilot trial, wider CIs are not unexpected. The use of a generalized estimating equation (GEE) and the corresponding adjustment is appropriate in situations where there are few clusters, though results are approximate and thus should be interpreted cautiously. This issue could be mitigated in larger trials with more clusters. The intervention did not have a specific component aimed at the antenatal period, though in retrospect it would be helpful to better prepare women for the introduction of new supportive care options. The use of the same clusters over a 54-week period means we cannot exclude the possibility that some women may have given birth twice during the study. We measured women’s experiences using a survey instrument in their language of choice; however, their responses may have been affected by social or courtesy biases.

Findings from this multicentered, stepped-wedge, cluster-randomized pilot trial suggest that the LCG strategy is a promising intervention that can improve quality of labor and childbirth care, reducing overuse of intrapartum interventions. This study provides important evidence on the debate around the introduction of the LCG into routine clinical practice internationally. Further evaluation in larger-scale, definitive trials are warranted.

## Methods

### Overview of study design

We designed and conducted a pragmatic, stepped-wedge, cluster-randomized pilot trial that was conducted between 1 July 2021 and 15 July 2022. We used an evidence-based, theory-informed approach to develop the intervention, and conducted the trial to determine whether it might have an effect on overuse of cesarean section or other important maternal and newborn outcomes. The trial was preceded by a 6-month formative phase, which was guided by the COM-B model of behavior change, which recognizes that individuals must have capability (C), physical and social opportunity (O) and motivation (M) to perform a behavior (B)^34^. We used co-design principles and primary data collection to develop and refine the ‘LCG strategy’ intervention, which included provider training and audit and feedback activities, and developed a theory of change (Supplementary Fig. 1). The intervention was then introduced in a stepwise manner in four public hospitals in the state of Karnataka, India, in accordance with a randomization schedule. Given the risk of cross contamination, individual randomization was not possible. We used a stepped-wedge approach as the LCG reflects the WHO’s current advice regarding standard of care^17^, and it was thus not ethically feasible to use a parallel-group design.

### Trial approvals and oversight

This trial was designed and conducted in accordance with the ethical principles of the World Medical Association’s Declaration of Helsinki, the Ottawa Statement for the Ethical Design and Conduct of Cluster Randomized Trials, and Good Clinical Practice standards^35,36,37^. We developed the trial protocol and reported findings in accordance with Standard Protocol Items: Recommendations for Interventional Trials (SPIRIT) guidance for randomized trials, and the Consolidated Standards of Reporting Trials (CONSORT) statement for stepped-wedge cluster-randomized trials (CONSORT checklist in Supplementary File 1)^38,39^. The trial protocol was preregistered (CTRI/2021/01/030695), with the protocol and statistical analysis plan published before trial closure; there were no major deviations or changes^40^.

We sought permission from the head of study hospitals (gatekeepers) and individual providers before commencing the trial. The study protocol specified a waiver of individual consent for data collected on women giving birth; these data were nonidentifiable, routinely collected clinical variables in medical records and labor ward registries. Routine medical records in participating hospitals, from which study data were captured, use the variable ‘sex’. For study participants invited to complete a postpartum survey, an informed consent was conducted. The trial was approved by the Alfred Hospital Human Ethics Committee (737/20), and the institutional ethics committees of the KLE Academy of Higher Education and Research (D-281120003), JJM Medical College, Davanagere (IEC-136/2020), Vijayanagar Institute of Medical Sciences (SVN IEC/20/2020-2021) and the Gadag Institute of Medical Sciences (IEC/01/2020-21), as well as the State Ethics Committee, Department of Health and Family Welfare, Government of Karnataka (DD(MH)/71/2020-21) and the Health Ministry’s Screening Committee, Indian Council of Medical Research (2020-10127). An independent, three-member Data and Safety Monitoring Committee oversaw the trial.

### Setting and participating healthcare providers

We purposively selected four public maternity hospitals in the state of Karnataka to participate. Eligibility criteria for these facilities were their capacity to provide comprehensive emergency obstetric care (including access to cesarean section), attending to more than 4,000 women giving birth each year, and having an overall cesarean section rate of 30% or more. In three hospitals, labor monitoring and partograph completion is primarily performed by postgraduate resident doctors, while in the remaining hospital it was performed by nurses. All hospitals had either completed or were undergoing accreditation under the Government of India’s national Labour Room Quality Initiative (‘LaQshya’), which is closely aligned with WHO intrapartum care recommendations^33^.

Each hospital was treated as a cluster. Two senior obstetricians working at each hospital were appointed as facility investigators and were responsible for trial activities at their hospital. The targets of the intervention were labor ward staff, including obstetricians, postgraduate doctors and nurses. These staff usually use a WHO simplified partograph to make decisions about labor interventions. We hypothesized that the intervention would promote correct LCG use by these providers, changing their labor monitoring and management practices to align with the WHO’s intrapartum recommendations. In turn, this could reduce overuse of cesarean section, improve maternal and newborn outcomes, and enhance women’s care experiences.

### Inclusion and exclusion criteria

The eligibility criteria for women to be in the study population were those giving birth at ≥20 weeks’ gestation in participating hospitals, during the study period. Pregnant women who were admitted but did not give birth at these hospitals were not included, nor were women who gave birth at another facility or in the community and arrived at a study hospital postpartum. The period of interest for study data collection was the time of a woman’s admission for childbirth until the time of discharge, transfer, death or until 7 days after admission (whichever came first).

### Randomization and blinding

Before trial commencement, the four clusters (hospitals) were randomly assigned to one of four sequences (H1, H2, H3 or H4; Fig. 1) using a computer-generated list of random numbers that was managed by the study statistician. The allocation sequence was concealed from the investigators and study teams and only revealed by the statistician 1 month before crossover to allow time for planning LCG strategy implementation activities. Once the hospital had commenced the intervention, blinding of hospital staff, research staff and individual women was not possible. The intervention was commenced in hospitals according to the randomly assigned sequence, with one hospital transitioning to intervention at 2-month intervals (that is, a step occurred every 2 months). A 2-week transition period was used to allow for the intervention to be fully adopted.

### Control and intervention

The control condition for the trial was current labor monitoring and management practices (‘usual clinical care’). While the WHO simplified partograph is widely used in India, the formative phase showed that its use was inconsistent and oftentimes retrospective. Training seminars were conducted at all hospitals on using the WHO simplified partograph to standardize the control condition. The WHO intrapartum care recommendations were also disseminated at all hospitals at the start of the trial.

The LCG strategy intervention was applied at cluster (hospital) level, and thus might affect all women giving birth in participating hospitals. The intervention included a co-designed LCG training program for doctors and nurses working on labor wards, and a monthly audit and feedback process using hospital cesarean section data (Supplementary File 2). For training, we developed and ran 2-day workshops for all labor ward staff, coordinated by facility investigators who had undergone a ‘training of trainers’ workshop. These workshops were based on WHO recommendations and the LCG manual^17,22^ and included practice clinical cases. After this, all providers working on labor wards underwent an 8-week ‘low-dose, high-frequency’ training program^41^, comprising clinical cases and bedside teaching using the LCG with senior clinical staff. The 8-week training was delivered in cycles to accommodate postgraduate resident rotations every 3 months. Refresher training was used if new staff arrived during the intervention period. All training activities encouraged providers to implement all aspects of the LCG, including offering or encouraging women on supportive care measures (labor companionship, pain relief options, oral intake, mobility, birth position of choice). At the time of randomization, all simplified WHO partographs in the labor ward were replaced with the LCG. Senior labor ward staff were encouraged to monitor and promote consistent, accurate LCG use through supportive supervision.

The intervention also included monthly audit and feedback meetings on cesarean rates using the Robson classification. Audit and feedback is widely used to promote evidence-based clinical practice, and is recommended by the WHO for avoiding unnecessary cesarean sections^26,42^. The WHO also recommends that countries use the Robson classification for assessing, monitoring and comparing their cesarean rates over time^9^. The Robson classification organizes all births in a facility into one of ten mutually exclusive, all-inclusive groups, on the basis of parity, previous cesarean, onset of labor, fetal presentation and lie, number of neonates and gestational age (term or preterm)^24^. Providers at randomized hospitals underwent a brief training based on the WHO implementation manual on how to interpret Robson classification data and how audit and feedback can help optimize cesarean section use. Robson classification tables were prepared using trial data and were shared directly with the study hospital on a monthly basis. These data were presented by senior clinical staff at monthly meetings, with structured discussions among the attendees on how to improve hospital performance. Hospitals and staff were instructed that all other aspects of clinical care during the trial should be in accordance with relevant local guidelines and protocols. In addition, facility leads were encouraged to identify and address anticipated barriers to the LCG strategy in their hospital. This included revision of hospital policies, standardization of clinical protocols, rearrangements to the physical labor ward environment and addressing some supply and equipment constraints. We used logbooks, tracking sheets and site visits to confirm that all eligible staff underwent LCG training activities, were using the LCG routinely and attended monthly cesarean audit meetings as planned.

### Primary and secondary outcomes

Trained research staff collected nonidentifiable, individual-level data on all eligible women (that is, those giving birth from 20 weeks’ gestation onwards) and their babies. Data were collected from the time of admission for childbirth until the time of discharge, transfer, death or until 7 days after admission (whichever came first).

The primary trial outcome was the use of cesarean section among women in Robson Group 1. Robson Group 1 is comprised of women who were nulliparous, gave birth to a singleton, term pregnancy in cephalic presentation and were in spontaneous labor. It is a subset of the obstetric population (usually around 30%) and includes largely low-risk women. The WHO advises that cesarean rates at or below 10% are achievable for Robson Group 1, while maintaining good outcomes^24^. However, in some low- and middle-income countries, the cesarean rate in Robson Group 1 exceeds 20% to 25%, indicative of overuse^43^. We anticipated that effects of the LCG strategy would be most easily detected in Robson Group 1. Conversely, the LCG strategy is unlikely to reduce cesarean use in higher-risk women, such as those with multiple pregnancies (Group 8) or with an oblique lie (Group 9), for whom the cesarean section rate is necessarily high. We did not anticipate any effects on antepartum cesarean use, as these women do not experience labor and thus do not require an LCG or partograph.

Secondary outcomes included use of intrapartum interventions (cesarean section, augmentation, artificial rupture of membranes, episiotomy, operative vaginal birth), maternal, fetal and neonatal mortality and severe morbidity outcomes, hospital admission and use of advanced newborn care interventions. The denominator varied depending on the outcome of interest (see Supplementary Table 1 for outcome definitions). We also measured women’s experiences of care using a pretested, interviewer-administered survey, conducted in a local language (Kannada, Hindi or Marathi), which was completed by postnatal day 7 or discharge (whichever came first) in a sample of postpartum women. This sample comprised women in Robson Group 1 or 3 who gave birth in the last 15 days of each 2-month period, had a liveborn baby, were 18 years or older and provided informed consent. In each hospital, trained interviewers approached and invited all eligible women to complete the survey.

All data were collected into predesigned study forms and managed using REDCap electronic data capture via tablets. Each hospital team had access to their own hospital data only, and facility investigators were responsible for checking completeness and accuracy of all collected data. To minimize errors, data validation processes were implemented in the data collection system. Statistical methods and data cleaning algorithms were utilized to identify potential errors and outliers for further investigation and correction. Regular data and trial progress review meetings and audits were conducted to identify and rectify any inconsistencies or outliers. Data monitors periodically visit the study sites to verify the accuracy and completeness of the collected data. They also provided training and guidance to study personnel, addressing any issues or concerns that might arise during the study. The trial concluded when 15 July 2022 was reached, as planned.

### Sample size

At the time of writing the trial protocol, no previous trial using the LCG had been conducted, meaning the effect size of our strategy was difficult to estimate. For the year 2020 (before the trial), these four hospitals collectively averaged 24,000 births per year, and their overall cesarean rate was 44%. The cesarean rate in women in Robson Group 1 (that is, the primary outcome) for all four hospitals was at least 40%. The trial was designed to provide 92% power to detect a 25% reduction in the Robson Group 1 cesarean rate from 40% to 30%, assuming an ICC equal to 0.02, a cluster auto correlation equal to 0.90 and an average of 300 women per cluster per time period with a coefficient of variation of cluster size equal to 0.60 (ref. ^44^).

### Statistical methods and analysis

Analyses were performed according to the intention-to-treat principle (according to planned exposure). Maternal baseline characteristics were summarized by trial arm as means and standard deviations or numbers and percentages, as appropriate. For the primary and secondary outcomes, a GEE to estimate the effect of the intervention with respect to the population average was used. A bias correction method and degree of freedom approximation due to the small number of clusters was applied in the GEE models to maintain the validity of the estimations. A Manck and DeRouen correction method with N-2 degrees of freedom was selected due to being the most conservative option^45^. An exchangeable correlation structure was assumed and the modified Poisson distribution with a log link function was considered. The model was constructed considering two variables: a binary indicator for treatment—indicating whether the observation was made during the control or the intervention period—and a categorical variable indicating the time period. The RR and the 95% CI were reported as the size effect. For the secondary outcomes, in which duration was measured in days, the effect size was calculated as the difference between the mean of days in the intervention group and the mean of days in the control group. The ICC was estimated under the control period using the GEE model. As no adjustment for multiplicity testing of secondary outcomes was considered, their results are reported as point estimates with 95% CIs and *P* values.

### Ethics and inclusion statement

Our study team support the principles of the Cape Town Statement, in particular the commitment to equitable international collaborations. The study was designed in partnership between three research groups (India, Argentina, Australia), building on multiple years of research collaborations and coauthored publications between several coauthors. This study was funded by a Global Grand Challenges grant—the submission was jointly prepared by J.P.V., S.G., Y.P., S.S.V., V.P., F.A. and L.G. This grant funding went to all three of our research organizations, with the largest amount of this funding received by the JNMC-India research team. The study protocol had 14 named investigators—12 from India, one from Argentina and one from Australia. J.P.V. and S.G. were named as co-Principal Investigators. During the study, decisions were taken by consensus among the steering group during fortnightly teleconferences. The authorship group (29 individuals) comprised 17 women and 12 men, and included late-, mid- and early-career individuals. Members of the authorship group include researchers in India (Y.P., S.S.V., M.S., S.B., J.A.K., S.B.P., A.K., R.R.A., P.M.R., S.S., L.B., M.H.M., S.S.G., S.C., B.R.), Argentina (V.P., F.A., L.G., M.B., A.C., R.R.) and Australia (J.P.V., E.A., C.S.E.H.). The lead author (J.P.V.) is in Australia and the senior author (S.G.) is in India. Our Technical Advisory Group (T.L., P.K., G.J.H., R.D.) included senior researchers from India, the United Kingdom, South Africa and the United States, and our Data and Safety Monitoring Committee included individuals from India, Switzerland and the United States.

### Reporting summary

Further information on the research design is available in the Nature Portfolio Reporting Summary linked to this article.

## Online content

Any methods, additional references, Nature Portfolio reporting summaries, source data, extended data, supplementary information, acknowledgements, peer review information; details of author contributions and competing interests; and statements of data and code availability are available at 10.1038/s41591-023-02751-4.

### Supplementary information

Supplementary Information Supplementary Files 1 and 2, Tables 1–4 and Fig. 1.

Reporting Summary

## Data Availability

In keeping with the Bill & Melinda Gates Foundation Open Access Policy, the de-identified individual-level data and the data dictionary are hosted publicly at the Gates Open Research-approved repository Zenodo. They can be accessed under 10.5281/zenodo.8140454. No restrictions on the availability of the data have been set.

## Code availability

R code used for data analysis along with detailed instructions on its usage is publicly available at 10.5281/zenodo.8140454. No restrictions on the availability of the code have been set.
