# Supplementary material for: Effects of the WHO Labour Care Guide on cesarean section in India: a pragmatic, stepped-wedge, cluster-randomized pilot trial
Source: Nat Med. 2024 Jan 30;30(2):463–9. doi: 10.1038/s41591-023-02751-4 (PMC10878967; doi:10.1038/s41591-023-02751-4)
Supplement: Supplementary file 1 — Supplementary Files 1 and 2, Tables 1–4 and Fig. 1. [file 41591_2023_2751_MOESM1_ESM.pdf]

nature medicine

Article https://doi.org/10.1038/s41591-023-02751-4

Effects of the WHO Labour Care Guide on cesarean section in India: a pragmatic, stepped-wedge, cluster-randomized pilot trial

In the format provided by the authors and unedited

Supplementary materials 3: Checklist of information to include when reporting a stepped wedge cluster randomised trial (SW-CRT)

Topic	Item no	Checklist item	Page no
**Title and abstract**			
	1a	Identification as a SW-CRT in the title.	1
	1b	Structured summary of trial design, methods, results, and conclusions (see separate SW-CRT checklist for abstracts).	2
**Introduction**			
Background and objectives	2a	Scientific background. Rationale for using a cluster design and rationale for using a stepped wedge design.	4
	2b	Specific objectives or hypotheses.	4
**Methods**			
Trial design	3a	Description and diagram of trial design including definition of cluster, number of sequences, number of clusters randomised to each sequence, number of periods, duration of time between each step, and whether the participants assessed in different periods are the same people, different people, or a mixture.	21
	3b	Important changes to methods after trial commencement (such as eligibility criteria), with reasons.	N/A
Participants	4a	Eligibility criteria for clusters and participants.	5-6
	4b	Settings and locations where the data were collected.	5
Interventions	5	The intervention and control conditions with sufficient details to allow replication, including whether the intervention was maintained or repeated, and whether it was delivered at the cluster level, the individual participant level, or both.	6
Outcomes	6a	Completely defined prespecified primary and secondary outcome measures, including how and when they were assessed.	7-8; 22-2
	6b	Any changes to trial outcomes after the trial commenced, with reasons.	N/A
Sample size	7a	How sample size was determined. Method of calculation and relevant parameters with sufficient detail so the calculation can be replicated. Assumptions made about correlations between outcomes of participants from the same cluster. (see separate checklist for SW-CRT sample size items).	8
	7b	When applicable, explanation of any interim analyses and stopping guidelines.	N/A
**Randomisation**			
Sequence generation	8a	Method used to generate the random allocation to the sequences of treatments.	6
	8b	Type of randomisation; details of any constrained randomisation or stratification, if used.	6
Allocation concealment mechanism	9	Specification that allocation was based on clusters; description of any methods used to conceal the allocation from the clusters until after recruitment.	6
Implementation	10a	Who generated the randomisation schedule, who enrolled clusters, and who assigned clusters to sequences.	6
	10b	Mechanism by which individual participants were included in clusters for the purposes of the trial (such as complete enumeration, random sampling; continuous recruitment or ascertainment; or recruitment at a fixed point in time), including who recruited or identified participants.	5; 8
	10c	Whether, from whom and when consent was sought and for what; whether this differed between treatment conditions.	5; 8
Blinding	11a	If done, who was blinded after assignment to sequences (eg, cluster level participants, individual level participants, those assessing outcomes) and how.	6
	11b	If relevant, description of the similarity of treatments.	6
Statistical methods	12a	Statistical methods used to compare treatment conditions for primary and secondary outcomes including how time effects, clustering and repeated measures were taken into account.	8-9
	12b	Methods for additional analyses, such as subgroup analyses, sensitivity analyses, and adjusted analyses.	N/A

(*Continued*)

Supplementary materials 3 (*Continued*)

Topic	Item no	Checklist item	Page no
**Results**			
Participant flow (a diagram is strongly recommended)	13a	For each treatment condition or allocated sequence, the numbers of clusters and participants who were assessed for eligibility, were randomly assigned, received intended treatments, and were analysed for the primary outcome (see separate SW-CRT flow chart).	9; 21
	13b	For each treatment condition or allocated sequence, losses and exclusions for both clusters and participants with reasons.	9; 21
Recruitment	14a	Dates defining the steps, initiation of intervention, and deviations from planned dates. Dates defining recruitment and follow-up for participants.	6; 21
	14b	Why the trial ended or was stopped.	8
Baseline data	15	Baseline characteristics for the individual and cluster levels as applicable for each treatment condition or allocated sequence.	9; 17
Numbers analysed	16	The number of observations and clusters included in each analysis for each treatment condition and whether the analysis was according to the allocated schedule.	9; 21
Outcomes and estimation	17a	For each primary and secondary outcome, results for each treatment condition, and the estimated effect size and its precision (such as 95% confidence interval); any correlations (or covariances) and time effects estimated in the analysis.	9-10; 18-
	17b	For binary outcomes, presentation of both absolute and relative effect sizes is recommended.	9-10, 18-
Ancillary analyses	18	Results of any other analyses performed, including subgroup analyses and adjusted analyses, distinguishing prespecified from exploratory.	N/A
Harms	19	Important harms or unintended effects in each treatment condition (for specific guidance see CONSORT for harms).	199-10;
**Discussion**			
Limitations	20	Trial limitations, addressing sources of potential bias, imprecision, and, if relevant, multiplicity of analyses.	12
Generalisability	21	Generalisability (external validity, applicability) of the trial findings. Generalisability to clusters or individual participants, or both (as relevant).	11
Interpretation	22	Interpretation consistent with results, balancing benefits and harms, and considering other relevant evidence.	10-11
**Other information**			
Registration	23	Registration number and name of trial registry.	5
Protocol	24	Where the full trial protocol can be accessed, if available.	5
Funding	25	Sources of funding and other support (such as supply of drugs), and the role of funders.	12
Research ethics review	26	Whether the study was approved by a research ethics committee, with identification of the review committee(s). Justification for any waiver or modification of informed consent requirements.	5

This checklist has been taken from table 3 in *BMJ* 2018;363:k1614, as a standalone document for readers to print out or fill in electronically.

Supplementary Table S1. Primary and secondary outcomes

Primary Outcome CS rate amongst women in Robson Group 1 (i.e. women who are nulliparous, singleton, cephalic, ≥37 weeks' gestation, in spontaneous labour). The numerator are the women in Robson Group 1 who had a CS and the denominator the number of women in Robson group 1.

Maternal Secondary Outcomes

Outcome	Outcome definition
CS rate in women in Robson Groups 1 and 3	Numerator: Number of women undergoing CS<br>Denominator: Number of women in Robson Groups 1 and 3
CS rate in women in Robson Groups 1 to 5	Numerator: Number of women undergoing CS<br>Denominator: Number of women in Robson Groups 1 to 5
Overall CS rate	Numerator: Number of women undergoing CS<br>Denominator: Number of women giving birth
Augmentation with oxytocin during labour rate	Numerator: Number of women given oxytocin for augmentation during labour<br>Denominator: Number of women who experienced spontaneous labour
Artificial rupture of the membranes rate	Numerator: Number of women who had artificial rupture of membranes<br>Denominator: Number of women who experienced spontaneous labour
Episiotomy rate	Numerator: Number of women who had episiotomy<br>Denominator: Number of women with vaginal birth
Operative vaginal birth rate	Numerator: Number of women who had operative vaginal birth (forceps or vacuum)<br>Denominator: Number of women with vaginal birth
Days between admission to childbirth	Mean of the days between admission to childbirth
Days between childbirth to discharge	Mean of the days between childbirth to discharge
3rd or 4th degree tears	Numerator: Number of women experiencing 3rd or 4th degree tears<br>Denominator: Number of women giving birth
PPH requiring uterine balloon tamponade or surgical intervention	Numerator: Number of women requiring uterine balloon tamponade OR surgical intervention for PPH<br>Denominator: Number of women giving birth
Suspected or confirmed maternal infection requiring therapeutic antibiotics	Numerator: Number of women with clinical signs or symptoms of maternal infection AND therapeutic antibiotics were required<br>Denominator: Number of women giving birth

Fetal/Neonatal Secondary Outcomes

Outcome	Outcome definition
Stillbirth	Numerator: Fetal death<br>Denominator: All born babies
Antepartum stillbirth	Numerator: Fetal death prior to admission<br>Denominator: All born babies
Intrapartum stillbirth	Numerator: Fetal death after admission<br>Denominator: All born babies
Apgar score <7 at 5 minutes	Numerator: Liveborn babies with Apgar <7 at 5 minutes<br>Denominator: Liveborn babies
Bag and mask ventilation of newborn	Numerator: Use of continuous bag and mask ventilation of newborn for >1 minute<br>Denominator: Liveborn babies
Mechanical ventilation of newborn	Numerator: Use of mechanical ventilation of newborn<br>Denominator: Liveborn babies
Composite neonatal outcome	Numerator: Use of mechanical ventilation of newborn or admission to NICU for suspected or confirmed or neonatal death

	Denominator: Liveborn babies
Prolonged (>48 hour) admission in NICU	Numerator: Admission to NICU for >48 hours<br>Denominator: Liveborn babies
Newborns requiring NICU admission for hypoxic ischaemic encephalopathy	Numerator: Admission to NICU for suspected or confirmed<br>Denominator: Liveborn babies
Composite neonatal outcome	Numerator: Use of mechanical ventilation of newborn or admission to NICU for suspected or confirmed or neonatal death<br>Denominator: Liveborn babies
Neonatal death	Numerator: Neonatal death in a liveborn infant by day 7 or discharge (whichever came first)<br>Denominator: All liveborn babies
Perinatal death	Numerator: Fetal death or neonatal death in a liveborn infant by day 7 or discharge (whichever came first)<br>Denominator: All born babies

Women's experience outcomes

Outcome	Outcome definition
Woman's experience with labour companion	Numerator: Women who reported a labour companion was present during labour or birth<br>Denominator: Women in Robson Group 1 or 3 who completed the survey
Woman's experience of being offered pain relief	Numerator: Women who reported that they were asked whether they would like any pain relief<br>Denominator: Women in Robson Group 1 or 3 who completed the survey
Women's satisfaction with their pain management during labour and birth	Numerator: Women who reported being very satisfied or somewhat satisfied with how their pain was managed during labour and birth<br>Denominator: Women in Robson Group 1 or 3 who completed the survey
Woman's experience of being encouraged to drink oral fluids	Numerator: Women who reported that a health worker encouraged them to drink water<br>Denominator: Women in Robson Group 1 or 3 who completed the survey
Woman's experience of being encouraged to eat food	Numerator: Women who reported that a health worker encouraged them to eat food<br>Denominator: Women in Robson Group 1 or 3 who completed the survey
Woman's experience of mobilising during labour	Numerator: Women who reported that a health worker encouraged them to walk around during labour<br>Denominator: Women in Robson Group 1 or 3 who completed the survey
Woman's experience of birth position of choice	Numerator: Women who reported that a health worker asked them which birth position they preferred<br>Denominator: Women in Robson Group 1 or 3 who completed the survey
Woman's experience of time health worker spent with them	Numerator: Women who reported being very satisfied or somewhat satisfied with amount of time health worker spent with them during labour<br>Denominator: Women in Robson Group 1 or 3 who completed the survey

Women's experience outcomes (cont.)

Outcome	Outcome definition
Women's satisfaction with the way health providers communicated with them	Numerator: Women who reported being very satisfied or somewhat satisfied with the way health workers communicated with them during labour and birth<br>Denominator: Women in Robson Group 1 or 3 who completed the survey
Woman's experience of privacy	Numerator: Number of women who strongly agreed or agreed that their privacy was respected during examinations and treatments<br>Denominator: Women in Robson Group 1 or 3 who completed the survey
Women's experience of being asked permission	Numerator: Number of women who said their health worker always asked permission before examinations and treatments<br>Denominator: Women in Robson Group 1 or 3 who completed the survey
Woman's overall experience of care	Numerator: Number of women who strongly agreed or agreed that they felt satisfied with their labour and birth experience<br>Denominator: Women in Robson Group 1 or 3 who completed the survey

Supplementary Table S2. Application of Robson Classification to intervention and control groups

Robson Classification Group	Intervention period (N = 14,814 women)	Control period (N = 11,517 women)
Group 1: Nulliparous, singleton, cephalic, term, spontaneous labour	4,302 (29.0%)	3,543 (30.8%)
Group 2: Nulliparous, singleton, cephalic, term, induced/prelabour Caesarean	1,729 (11.7%)	1,022 (8.9%)
• Group 2a: Nulliparous, singleton, cephalic, term, induced	848 (5.7%)	471 (4.1%)
• Group 2b: Nulliparous, singleton, cephalic, term, prelabour Caesarean	881 (5.9%)	551 (4.8%)
Group 3: Multiparous (no previous Caesarean), singleton, cephalic, term, spontaneous labour	3,183 (21.5%)	2,661 (23.1%)
Group 4: Multiparous (no previous Caesarean), singleton, cephalic, term, induced/prelabour Caesarean	450 (3.0%)	282 (2.4%)
• Group 4a: Multiparous (no previous Caesarean), singleton, cephalic, term, induced	292 (2.0%)	212 (1.8%)
• Group 4a: Multiparous (no previous Caesarean), singleton, cephalic, term, prelabour Caesarean	158 (1.0%)	70 (0.6%)
Group 5: Previous Caesarean, singleton, cephalic, term, (spontaneous labour, induced labour or prelabour Caesarean)	3,071 (20.7%)	2,300 (20.0%)
Group 6: Nulliparous with a singleton breech	235 (1.6%)	182 (1.6%)
Group 7: Multiparous with a singleton breech (including previous Caesarean)	224 (1.5%)	153 (1.3%)
Group 8: Multiple pregnancies (including previous Caesarean)	155 (1.0%)	107 (0.9%)
Group 9: Single pregnancy, transverse or oblique lie (including previous Caesarean)	21 (0.1%)	36 (0.3%)
Group 10: Singleton, cephalic, preterm (including previous Caesarean)	1,444 (9.7%)	1,231 (10.7%)

Supplementary Table S3. Serious adverse events by period

	Intervention Period (N of women = 14,814) (N of liveborns=14,522) (N of newborns=14,971)	Transition period (N of women=1,080) (N of liveborns=1,060) (N of newborns: 1,089)	Control Period (N of women= 11,517) (N of liveborns= 11,257) (N of newborns= 11,624)
	n (%)	n (%)	n (%)
Maternal death	13 (0.09)	1 (0.09)	5 (0.04)
Neonatal death	200 (1.38)	11 (1.04)	196 (1.74)
Neonatal death (less than 28 weeks)	18 (0.12)	1 (0.09)	16 (0.14)
Neonatal death (28 weeks or more)	182 (1.25)	10 (0.94)	180 (1.60)
Stillbirth	449 (3.00)	29 (2.66)	367 (3.16)
Stillbirth (less than 28 weeks)	175 (1.17)	10 (0.92)	139 (1.20)
Stillbirth (28 weeks or more)	274 (1.83)	19 (1.74)	228 (1.96)

Supplementary Table S4. Causes of maternal deaths, by period

	Intervention Period (N = 13)	Transition Period (N = 1)	Control Period (N = 5)
Pre-eclampsia/eclampsia	5	0	4
Obstructed labour	0	0	0
Haemorrhage	1	0	0
Infection	2	1	0
Other*	5	0	1

*The case classified as "Other" in the control period was a postpartum cardiomyopathy. The five cases classified as "Other" in the intervention period were: (1) Immediate cause: a) Hepatic encephalopathy with MODS Antecedent cause: b) Acute fatty liver of pregnancy, (2) Amniotic fluid embolism, (3) Disseminated intravascular coagulation secondary to acute fatty liver of pregnancy, (4) Cerebrovascular Accident, (5) Pulmonary embolism.

Supplementary Figure S1. Diagram showing a ‘theory of change’ for the LCG strategy intervention

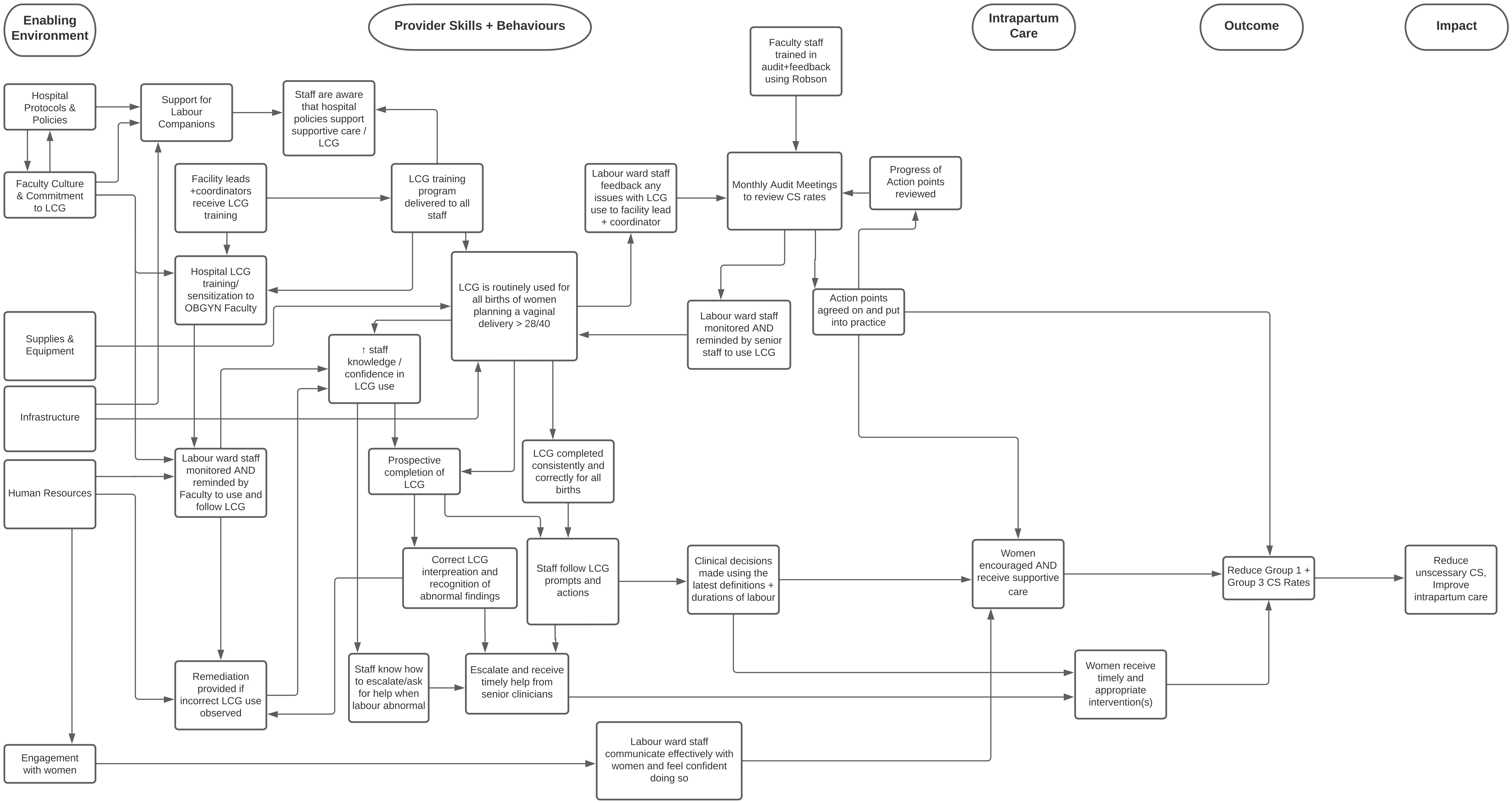

Supplementary File S2. Detailed description of intervention.

The trial intervention had two main components:

Part A - Implementing the LCG*:* The LCG is used by maternity care providers to monitor women during labour and childbirth and help promote best-practice intrapartum care. Providers underwent an initial standardised two-day training workshop using the WHO LCG Manual and a co-designed training package on how to use the LCG. The training was provided by senior obstetricians at the participating hospitals who had previously completed a “training of trainers” workshop, with support from a LCG master trainer.

Weekly case-based learning using LCG for labour ward providers was conducted for an 8-week period after the initial training workshop. This case-based learning program was based on the “low-dose, high-frequency” approach that has proved effective in recent maternal and newborn health education interventions.(22) Periodic refresher training for existing staff and additional training for new staff was conducted to ensure that all providers working in labour ward received adequate LCG training.

In addition, the introduction and routine use of LCG was communicated to staff through posters, checklists in medical records, and other staff awareness and engagement activities. Blank LCGs were widely available in labour ward (replacing simplified partographs) from time of randomization. Supportive supervision on LCG use by senior clinical staff to other health providers. By supportive supervision, we mean that clinical supervisors regularly observed labour ward staff completing the LCG in real time, provided constructive feedback and support, and ensured errors were corrected and queries resolved as they arose. This approach aimed to encourage open, two-way communication, facilitate problem-solving, and provide regular follow-up and review with staff by supervisors to ensure that appropriate intrapartum decision-making is being applied.

Part B - Audit and feedback using Robson Classification**:**
Audit and feedback is a widely used strategy to promote evidence-based practice, where clinical performance indicators are provided to healthcare providers to drive improvements. A 2012 Cochrane review of 140 studies using audit and feedback interventions concluded that they can improve clinical practice, though effects are often modest.(23) Factors associated with improved outcomes include: when the source of feedback is a supervisor, colleague or respected opinion leader; delivered at least monthly; provided on more than one occasion; is both verbal and written; includes explicit targets and an action plan.

In their 2018 guideline, WHO recommends that “implementation of evidence-based clinical practice guidelines, caesarean section audits and timely feedback to health-care professionals are recommended to reduce caesarean births”. This was on the basis of high-certainty evidence that showed that implementation of guidelines combined with audit and feedback could slightly reduce CS rates in women with low-risk pregnancies (-1.7% risk difference).(24) WHO also recommends that countries use the Robson Classification for assessing, monitoring and comparing their CS rates over time.(25) The Robson Classification organises all births in a facility into one of 10 mutually exclusive, all-inclusive groups, on the basis of parity, previous CS, onset of labour, fetal presentation, number of neonates and gestational age (term or preterm).(26)

Randomised hospitals were provided with a 2-hour online training workshop on how to interpret and classify CS data by Robson Classification and how to conduct audit and feedback sessions at their hospital sites. Robson Classification tables were prepared by an independent analyst based on data collected during the trial and shared directly with the study hospital on a monthly basis (from time of randomization until end of trial). The trial steering group was blinded to these reports. Hospital leads were responsible for organising audit meetings each month, where these Robson Classification tables were presented to labour ward staff at monthly meetings, with structured discussions on how to improve performance. There were opportunities for staff to reflect on their experience with the LCG and identify areas where they feel they need more improvement or support. These meetings were embedded within usual clinical meeting activities at the hospital and meeting minutes were taken by a delegated staff member.
