# Supplementary material for: Effects of the WHO Labour Care Guide on cesarean section in India: a pragmatic, stepped-wedge, cluster-randomized pilot trial
Source: Nat Med. 2024 Jan 30;30(2):463–9. doi: 10.1038/s41591-023-02751-4 (PMC10878967; doi:10.1038/s41591-023-02751-4)
Supplement: Supplementary file 2 — Reporting Summary [file 41591_2023_2751_MOESM2_ESM.pdf]

nature portfolio

Corresponding author(s): Joshua Vogel

Last updated by author(s): Nov 26, 2023

Reporting Summary

Nature Portfolio wishes to improve the reproducibility of the work that we publish. This form provides structure for consistency and transparency in reporting. For further information on Nature Portfolio policies, see our Editorial Policies and the Editorial Policy Checklist.

Statistics

For all statistical analyses, confirm that the following items are present in the figure legend, table legend, main text, or Methods section.

n/a	Confirmed	
☐	☒	The exact sample size (*n*) for each experimental group/condition, given as a discrete number and unit of measurement
☐	☒	A statement on whether measurements were taken from distinct samples or whether the same sample was measured repeatedly
☐	☒	The statistical test(s) used AND whether they are one- or two-sided<br>*Only common tests should be described solely by name; describe more complex techniques in the Methods section.*
☐	☒	A description of all covariates tested
☐	☒	A description of any assumptions or corrections, such as tests of normality and adjustment for multiple comparisons
☐	☒	A full description of the statistical parameters including central tendency (e.g. means) or other basic estimates (e.g. regression coefficient) AND variation (e.g. standard deviation) or associated estimates of uncertainty (e.g. confidence intervals)
☐	☒	For null hypothesis testing, the test statistic (e.g. F, t, r) with confidence intervals, effect sizes, degrees of freedom and P value noted<br>*Give P values as exact values whenever suitable.*
☒	☐	For Bayesian analysis, information on the choice of priors and Markov chain Monte Carlo settings
☐	☒	For hierarchical and complex designs, identification of the appropriate level for tests and full reporting of outcomes
☐	☒	Estimates of effect sizes (e.g. Cohen's *d*, Pearson's *r*), indicating how they were calculated

Our web collection on statistics for biologists contains articles on many of the points above.

Software and code

Policy information about availability of computer code

Data collection	REDCap 9.5.36
Data analysis	R version 4.1.1. - see https://doi.org/10.5281/zenodo.8140454 for analysis code

For manuscripts utilizing custom algorithms or software that are central to the research but not yet described in published literature, software must be made available to editors and reviewers. We strongly encourage code deposition in a community repository (e.g. GitHub). See the Nature Portfolio guidelines for submitting code & software for further information.

Data

Policy information about availability of data

All manuscripts must include a data availability statement. This statement should provide the following information, where applicable:

- Accession codes, unique identifiers, or web links for publicly available datasets
- A description of any restrictions on data availability
- For clinical datasets or third party data, please ensure that the statement adheres to our policy

The trial dataset generated during this study, and the data dictionary and syntax used for analyses (codes) are deposited hosted at the Gates Open Research-approved repository Zenodo under DOI: https://doi.org/10.5281/zenodo.8140454

Human research participants

Policy information about studies involving human research participants and Sex and Gender in Research.

Reporting on sex and gender	This was a trial of women giving birth in four hospitals, and we have used the term women throughout.
Population characteristics	Maternal age; history of Caesarean section; Gravida, Parity, receipt of antenatal care, gestational age at time of birth (See Table 1)
Recruitment	The eligibility criteria for women to be in the study population were those giving birth at >=20 weeks' gestation in participating hospitals, during the study period. Pregnant women who were admitted but did not give birth at these hospitals were not included, nor were women who gave birth at another facility or in the community and arrived at a study hospital postpartum. The period of interest for study data collection was the time of a woman's admission for childbirth until the time of discharge, transfer, death or until 7 days after admission (whichever came first). The study protocol specified a waiver of individual consent for data collected on women giving birth – these data were non-identifiable, routinely-collected clinical variables in medical records and labour ward registries
Ethics oversight	The trial protocol (including the consent waiver) was approved by the Alfred Hospital Human Ethics Committee (737/20), and the institutional ethics committees of the KLE Academy of Higher Education and Research (D-281120003), J J M Medical College, Davanagere (IEC-136/2020); Vijayanagar Institute of Medical Sciences (SVN IEC/20/2020-2021) and the Gadag Institute of Medical Sciences, (IEC/01/2020-21), as well as the State Ethics Committee, Department of Health and Family Welfare, Government of Karnataka (DD(MH)/71/2020-21); and the Health Ministry's Screening Committee, Indian Council of Medical Research (2020-10127).

Note that full information on the approval of the study protocol must also be provided in the manuscript.

Field-specific reporting

Please select the one below that is the best fit for your research. If you are not sure, read the appropriate sections before making your selection.

☐ Life sciences ☒ Behavioural & social sciences ☐ Ecological, evolutionary & environmental sciences

For a reference copy of the document with all sections, see nature.com/documents/nr-reporting-summary-flat.pdf

Behavioural & social sciences study design

All studies must disclose on these points even when the disclosure is negative.

Study description	a pragmatic, stepped-wedge, cluster-randomized trial
Research sample	The research sample were women giving birth at 20 weeks' gestation at later, in participating hospitals during the study period.<br><br>Pregnant women who were admitted but did not give birth at these hospitals were not included, nor were women who gave birth at another facility or in the community and arrived at a study hospital postpartum. The period of interest for study data collection was the time of a woman's admission for childbirth until the time of discharge, transfer, death or until 7 days after admission (whichever came first).<br><br>The rationale for this sample is that the intervention (LCG strategy) could potentially have an effect on women giving birth in the study hospitals. Women who did not give birth, or who had given birth elsewhere, could not have experienced any effect of the intervention.<br><br>As the eligibility criteria was broad, the sample is representative of women giving birth in the participating hospitals.<br><br>Collected data were non-identifiable, routinely-collected clinical variables in medical records and labour ward registries. Routine medical records in participating hospitals, from which study data were captured, use the variable 'sex' – on this basis, 100% of study participants were reported as 'women'. The mean age of women in the intervention group was 23.9 years (SD 3.6) and for the control group it was 23.4 years (SD 3.6).
Sampling strategy	This trial used a health outcome (CS rate in Robson Group 1) to evaluate the effects of the LCG strategy intervention. However, as this is a new, complex intervention that had not been previously evaluated, the effect size and intra-cluster correlation (ICC) was difficult to estimate prior to the trial.<br><br>In the year 2020, prior to trial commencement, the four hospitals collectively had on average approximately 24,000 births per year (around 4000 births every 2 months) and the overall CS rate across all hospitals was approximately 44%.<br><br>We estimated the CS rate in women in Robson Group 1 in these four hospitals to be at least 40%. This would mean that, across all

four hospitals, approximately 1300 women in Robson Group 1 would give birth every 2 months (i.e., an average of 325 women per cluster).

The trial was designed to provide 92% power, to detect a 25% reduction in the Robson Group 1 Caesarean rate from 40% to 30%. We assumed an intraclass correlation coefficient (ICC) equal to 0.02, a cluster auto correlation equal to 0.90, and an average of 300 women per cluster per time period with a coefficient of variation of cluster size equal to 0.60.

Data collection	As this was a stepped-wedge, cluster randomised trial involving a complex intervention, it was not possible to blind participants, providers or research staff. All research staff underwent a standardised training to understand the study aims, hypothesis, intervention and outcomes prior to commencing data collection.<br><br>The LCG strategy intervention included multiple healthcare provider training activities, and implementing monthly audit and feedback meetings at hospital level using Caesarean section data. Healthcare providers were thus the target of the LCG strategy intervention.<br><br>Trained research staff collected non-identifiable, individual-level data on all women giving birth from 20 weeks' gestation onwards and their babies. Data were collected from the time of admission for childbirth until the time of discharge, transfer, death or until 7 days after admission (whichever came first).<br><br>We also measured women's experiences of care using a pre-tested, interviewer-administered survey, conducted in a local language (Kannada, Hindi or Marathi), that was completed by postnatal day 7 or discharge (whichever came first) in a sample of postpartum women. This sample comprised women in Robson Group 1 or 3 who gave birth in the last 15 days of each 2-month period, had a liveborn baby, were 18 years or older, and who provided informed consent. In each hospital, trained interviewers approached and invited all eligible women to complete the survey. Healthcare providers were not present during the conduct of this survey.<br><br>All data were collected into pre-designed study forms and managed using REDCap electronic data capture via tablets.
Timing	Between 1 July 2021 and 15 July 2022
Data exclusions	The data collected during the transition period were excluded from the primary and secondary analyses (1,080 women and 1,089 babies) - this was pre-established and is consistent with analysis methodology for stepped-wedge, cluster-randomised trials.
Non-participation	No participants dropped out from the main trial, which captured data on all eligible women giving birth in participating hospitals. For the postpartum survey of a sub-sample of women, a total of 1,438 women in the control group and 1,277 women in the intervention group consented (100% and 99.9% consent rate, respectively) and completed postpartum surveys.
Randomization	Prior to trial commencement, the four clusters (hospitals) were randomly assigned to one of four sequences (H1, H2, H3, or H4, see Figure 1) using a computer-generated list of random numbers that was managed by the study statistician. The allocation sequence was concealed from the investigators and study teams and only revealed by the statistician one month prior to cross over to allow time for planning LCG implementation activities. Once the hospital had commenced the intervention, blinding of hospital staff, research staff and individual women was not possible. The intervention was commenced in hospitals according to the randomly assigned sequence, with one hospital transitioning to intervention at 2-month intervals (i.e., a step occurred every 2 months). A two-week transition period was used to allow for the intervention to be fully adopted.

Reporting for specific materials, systems and methods

We require information from authors about some types of materials, experimental systems and methods used in many studies. Here, indicate whether each material, system or method listed is relevant to your study. If you are not sure if a list item applies to your research, read the appropriate section before selecting a response.

Materials & experimental systems

n/a	Involved in the study	
☒	☐	Antibodies
☒	☐	Eukaryotic cell lines
☒	☐	Palaeontology and archaeology
☒	☐	Animals and other organisms
☐	☒	Clinical data
☒	☐	Dual use research of concern

Methods

n/a	Involved in the study	
☒	☐	ChIP-seq
☒	☐	Flow cytometry
☒	☐	MRI-based neuroimaging

Clinical data

Policy information about clinical studies

All manuscripts should comply with the ICMJE guidelines for publication of clinical research and a completed CONSORT checklist must be included with all submissions.

Clinical trial registration	CTRI/2021/01/030695
Study protocol	Published: https://reproductive-health-journal.biomedcentral.com/articles/10.1186/s12978-022-01525-4

Data collection	We purposively selected four public maternity hospitals in Karnataka State to participate, based on their capacity to provide comprehensive emergency obstetric care (including access to caesarean section). All four hospitals attend to more than 4,000 women giving birth each year, and have an overall caesarean section rate of 30% or more.<br><br>All data were collected during the study period by research staff based at these hospitals only.<br><br>Recruitment / data collection was conducted during the trial period only - 1 July 2021 to 15 July 2022.
Outcomes	The primary trial outcome was the use of Caesarean section amongst women in Robson Group 1. That is, women who were nulliparous, gave birth to a singleton, term pregnancy in cephalic presentation, and were in spontaneous labour.<br><br>Secondary outcomes included use of intrapartum interventions, and maternal, fetal and neonatal health outcomes. The denominator varied depending on the outcome of interest (see Supplementary Table S1 for outcome definitions).<br><br>We also measured women's experiences of care using a pre-tested, interviewer-administered survey, conducted in a local language (Kannada, Hindi or Marathi), that was completed by postnatal day 7 or discharge (whichever came first) in a sample of postpartum women.
